# LncRNA MIAT sponges miR-149-5p to inhibit efferocytosis in advanced atherosclerosis through CD47 upregulation

**DOI:** 10.1038/s41419-019-1409-4

**Published:** 2019-02-12

**Authors:** Zi-ming Ye, Shuai Yang, Yuan-peng Xia, Rui-ting Hu, Shengcai Chen, Bo-wei Li, Shao-li Chen, Xue-ying Luo, Ling Mao, Yanan Li, Huijuan Jin, Chao Qin, Bo Hu

**Affiliations:** 1 Department of Neurology, Union Hospital, Tongji Medical College, Huazhong University of Science and Technology, 430022 Wuhan, China; 2 Department of Neurology, The First Affiliated Hospital, Guangxi Medical University, Nanning, 530021 Guangxi China

## Abstract

Atherosclerotic cardio-cerebrovascular disease and death remain the leading cause of morbidity and mortality worldwide. Defective efferocytosis, the clearance of apoptotic cells by macrophages, is thought to lead to increased inflammation and necrotic core formation in atherosclerotic lesions. However, very little is known about the role of long noncoding RNA (lncRNA) during this process. Here we show that lncRNA myocardial infarction associated transcript (MIAT) was markedly elevated in the serum of patients with symptoms of vulnerable atherosclerotic plaque and the macrophages of necrotic cores in an advanced atherosclerosis mouse model. MIAT knockdown attenuated atherosclerosis progression, reduced necrotic core size, and increased plaque stability in vivo. Furthermore, MIAT knockdown promoted clearance of apoptotic cells by macrophages in vivo and in vitro. Mechanistic studies revealed that MIAT acted as a micro RNA (miRNA) sponge to positively modulate the expression of anti-phagocytic molecule CD47 through sponging miR-149-5p. Together, these findings identified a macrophage MIAT/miR-149-5p /CD47 pathway as a key factor in the development of necrotic atherosclerotic plaques.

## Introduction

Atherosclerotic plaque vulnerability has been identified as the major cause of the atherosclerotic cerebrovascular disease^1,2^. While the majority of atherosclerotic plaques remain clinically silent, some instable plaques may suddenly rupture after decades of indolent progression and cause life-threating acute ischemic events^3,4^. Therefore, the ability to identify and stabilize vulnerable plaques would be of great value in patients at high risk of lesion rupture. Long noncoding RNAs (lncRNAs) belong to a class of non-protein-coding RNAs longer than 200 nucleotides involved in the epigenetic regulation by modulating gene expression^5^. There is growing evidence implicating some atherosclerosis-related lncRNAs in plasma lipid homeostasis, such as cholesterol absorption, uptake of modified lipoproteins, and reverse cholesterol transport, thereby affecting the progression of atherosclerosis^6,7^. Moreover, recent studies have shown that lncRNAs exist in the circulation and can serve as independent biomarkers reflecting the local disease process in cardiovascular disease^8,9^. Further investigation into the roles of lncRNAs in atherosclerotic plaque progression and vulnerability, as well as the exact mechanisms involved are needed.

Myocardial infarction associated transcript (MIAT), also termed as Gomafu in humans or Rncr2 in mice, is a highly conserved mammalian lncRNA^10,11^. Many researchers have conducted studies and found that MIAT is involved in various physiological and pathological processes, including neuron development, formation of nuclear bodies, microvascular dysfunction, and myocardial infarction^11–13^. Recent reports based on the quantitative polymerase chain reaction (PCR) have indicated that MIAT is highly expressed in human carotid plaques and might be a potential diagnostic indicator in ischemic stroke^14,15^. These results suggest that MIAT may have potential roles in atherosclerotic cerebrovascular disease. Therefore, in the current study we aimed to determine whether MIAT was a critical regulator of plaque vulnerability and to define the underlying molecular mechanisms.

In atherosclerosis, the uptake of modified lipoproteins by macrophages exceeds cholesterol efflux, leading to the deposition of cholesterol esters and the subsequent formation of foam cells^16,17^. Apoptosis and the secondary necrosis of foam cells are thought to be major causes of necrotic core development and contribute toward the formation of vulnerable plaques^18,19^. Under physiological condition, these apoptotic cells are rapidly cleared by macrophages and other phagocytes through a process known as efferocytosis^20,21^. Recent results have revealed that efferocytosis is defective in advanced plaques of human and animals, which may explain why necrosis cells constantly accumulate in the necrotic core and aggravate the inflammatory response^18,22,23^. Efferocytosis is mediated by macrophages recognizing phagocytic ‘eat me’ signals on the apoptotic cells and can be counterbalanced by anti-phagocytic ‘don’t eat me’ signal such as the CD47 molecule^20,24^. Among the antiphagocytic signal molecules, CD47 has been identified as a novel therapeutic target for treating atherosclerosis by promoting efferocytosis^24^. However, previous studies reported that anti-CD47 antibody therapy contributed to splenic erythrophagocytosis and compensatory reticulocytosis^25,26^. In addition, it was found that the anti-CD47 antibodies are not curative alone, possibly due to the molecular weight of antibodies being so large and the limited tissue penetration^27^. Therefore, further studies are warranted to define additional measures to target the regulation of CD47 expression.

In the current study, we found that MIAT was significantly upregulated in serum of patients with symptoms of atherosclerotic vulnerable plaque and was consistently increased in serum and macrophages of necrotic cores in an advanced atherosclerosis mouse model. Subsequent knockdown of MIAT in vivo demonstrated that silencing of MIAT significantly enhanced phagocytic clearance and reduced atherosclerosis plaque progression and instability. Mechanistically, MIAT acted as a micro RNA (miRNA) sponge to positively modulate the expression of CD47 through sponging miR-149-5p. Therefore, our study provides new insights into the molecular function of the MIAT/miR-149-5p/CD47 signaling pathway in the pathogenesis of plaque vulnerability and highlights the potential of MIAT as a new therapeutic target for atherosclerosis.

## Results

### MIAT was upregulated in symptomatic human atherosclerotic disease and in advanced mouse atherosclerosis

**Fig. 1:**
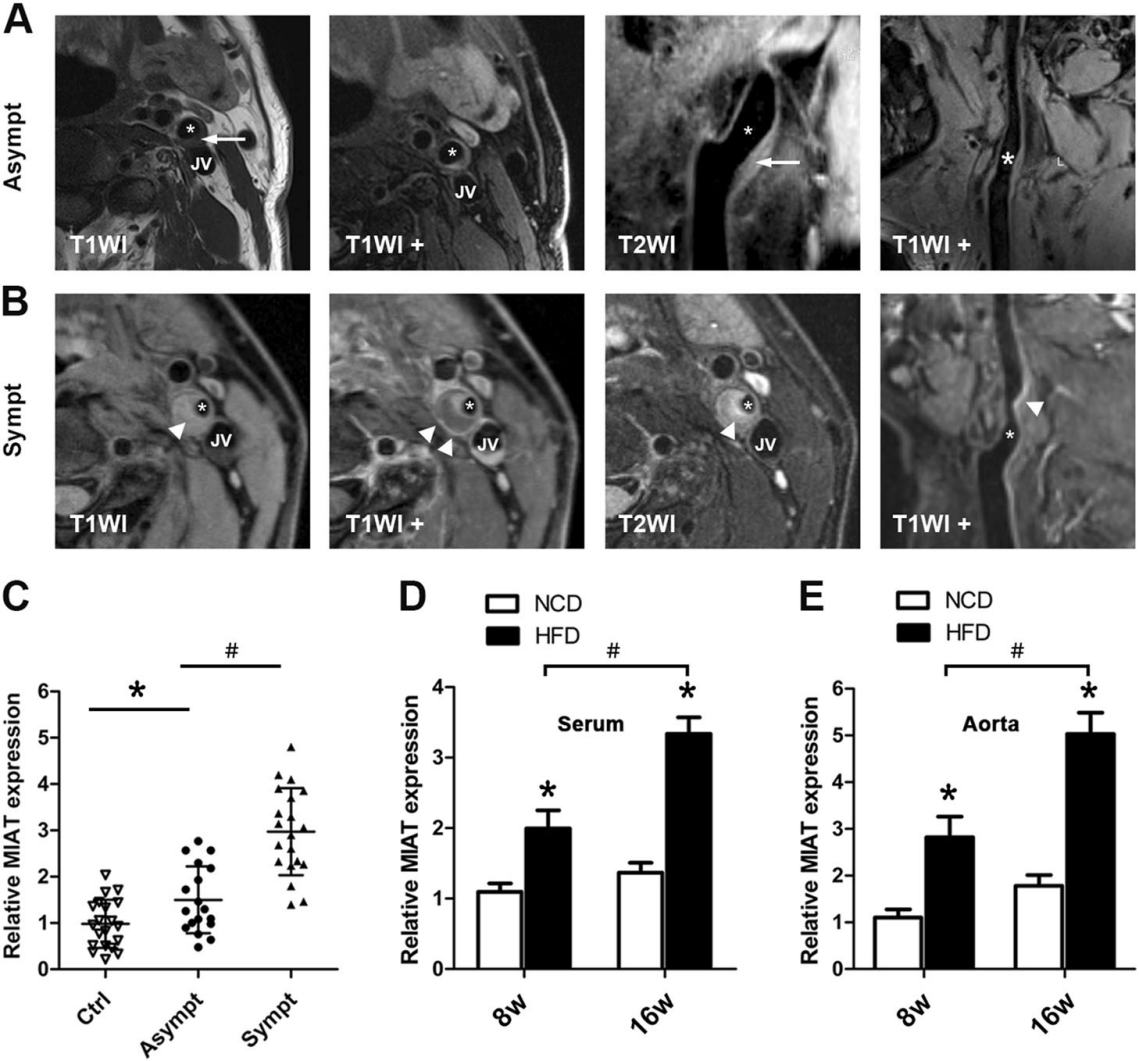
Expression of MIAT increased in symptomatic human atherosclerotic disease and in advanced mouse atherosclerosis. **a** Example of type III lesion in the internal carotid artery (extracellular lipid pool was detected by histology). On multicontrast-weighted MR images, iso-intensity on T1WI and slightly higher signal on T2WI (arrow), no enhancement in T1WI + . The asterisks (*) indicates lumen. JV indicates jugular vein. **b** Example of type IV–V lesion in the internal carotid artery (lipid-rich necrotic core was detected by histology). On multicontrast-weighted MR images, lipid-rich necrotic core (arrowheads) had slightly higher signal on both T1WI and T2WI and had enhancement in T1WI + (arrowheads). Lumen is severely stenosed. The asterisks (*) indicates lumen. **c** Quantitative reverse transcriptase-polymerase chain reaction (qRT-PCR) analysis of expression of MIAT in the serum from control (Ctrl, *n* = 20), asymptomatic (Asympt, *n* = 18) or symptomatic (Sympt, *n* = 20) subjects with recent TIA/stroke (*P<0.05, Asympt patients vs. Ctrl, ^#^*P*<0.05, Sympt patients vs. Asympt patients). **d**, **e** Analysis of MIAT expression in the serum and aorta of atherosclerotic lesions of HFD-Fed ApoE^−/−^ mice and NCD-fed ApoE mice^−/−^ at 8 and 16 weeks by qRT-PCR (*P < 0.05, HFD-fed ApoE^−/−^ mice vs. NCD-fed ApoE^−/−^ mice, ^*#*^*P*<0.05, HFD-fed ApoE^−/−^ mice at 16 week vs. HFD-fed ApoE^−/−^ mice at 8 week, *n* = 6/group)

Disease-associated lncRNAs can often be detected in the circulation and may biologically influence the local disease process^7,8^. We first assessed whether the expression of MIAT was altered in atherosclerotic disease using quantitative reverse transcription polymerase chain reaction (qRT-PCR) analysis of serum RNA extracted from the control group and atherosclerotic patients who had suffered either a transient ischemic attack or stroke (symptomatic) or were asymptomatic. In addition, to determine if the plaques in the atherosclerotic patients were stable or vulnerable, we used carotid magnetic resonance imaging (CMIR) to detect the subjects, CMIR is capable of accurately characterizing plaque morphology, composition, and surface condition and has been extensively validated by histology (Fig. 1a, b). We found that the serum of symptomatic subjects had higher levels of MIAT compared with those of asymptomatic patients and that serum MIAT levels were also increased in asymptomatic patients compared to those in the control group (Fig. 1c). The baseline and clinical characteristics of the study populations are shown in Supplementary Table 1. Comparison of some parameters including age, sex, and lipid profiles among the three groups showed no statistically significant differences. To further investigate the correlation of MIAT and atherosclerotic plaque progression, we detected its expression in ApoE^−/−^ mice fed a high-fat diet (HFD) for 8 weeks, which induced relatively early atherosclerotic lesions, and in mice fed HFD for 16 weeks, which resulted in the development of advanced plaques. As expected, the expression levels of MIAT in the serum in HFD-fed ApoE^−/−^ mice were ≈1.83-fold and ≈2.40-fold higher, respectively, than that in normal chow diet (NCD)-fed ApoE^−/−^ mice at 8 and 16 weeks (Fig. 1d). In addition, the expression levels of MIAT in the aorta in HFD-fed ApoE^−/−^ mice were ≈2.56-fold and ≈2.83-fold higher, respectively, than that in NCD-fed ApoE^−/−^ mice at 8 and 16 weeks (Fig. 1e). These results suggested that MIAT may be involved in the progression and instability of atherosclerotic plaques.

### MIAT was upregulated in macrophages of advanced atherosclerotic lesions and was induced in vitro by ox-LDL

**Fig. 2:**
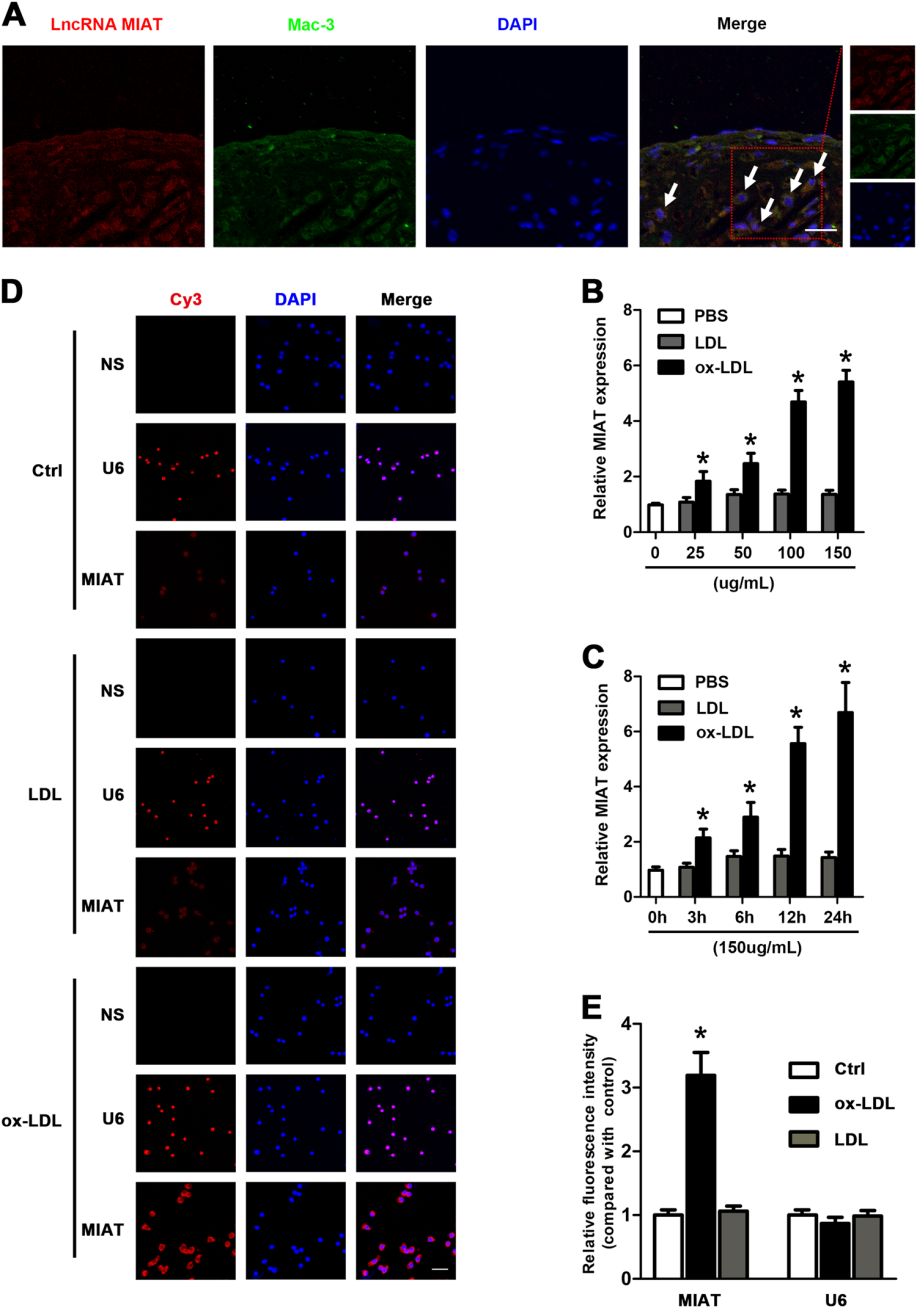
Expression of MIAT expressed in macrophages in advanced atherosclerotic lesions and its upregulation in macrophages after ox-LDL treatment. **a** RNA-fluorescent in situ hybridization (FISH) for MIAT (red) and immunostaining for Mac-3 (green) was performed on frozen aortic root sections from 16-week HFD-fed ApoE^−/−^ mice. Arrows denote macrophages expressing MIAT. (Scale bar = 50 μm). **b**, **c** qRT–PCR analysis of MIAT expression in ox-LDL or LDL-treated Raw264.7 cells at indicated dose (0, 25, 50, 100, 150 μg/mL) and at the indicated time (0, 3, 6, 12, 24 h) (*P<0.05 oxLDL vs. LDL, *n* = 6/group). **d, e** RNA-FISH for MIAT (red) in Raw264.7 cells after ox-LDL treatment. Nuclei, blue. U6 was detected as a positive control. (*P < 0.05 oxLDL vs. Ctrl, n = 6/group). Scale bar = 20 μm

To better understand the location and potential role of MIAT in advanced atherosclerosis, we performed RNA fluorescent in situ hybridization (RNA-FISH) on the advanced plaques. We found that the majority of the lesion macrophages expressed MIAT based on combined immunostaining for MIAT and Mac-3 (Fig. 2a). Additionally, a few smooth muscle cells (SMCs) also expressed MIAT (Supplementary Fig. 1). Furthermore, Raw264.7 cells were stimulated with oxidized low-density lipoprotein (ox-LDL) or native LDL. Expression levels of MIAT were significantly increased after ox-LDL treatment in dose-dependent and time-dependent manners (Fig. 2b, c). We also determined by in vitro RNA-FISH that MIAT was localized predominantly in the cytoplasm and not in the nucleus and that MIAT expression markedly increased after ox-LDL treatment (Fig. 2d, e).

### Knockdown of MIAT attenuated atherosclerosis progression in ApoE^−/−^ mice

**Fig. 3:**
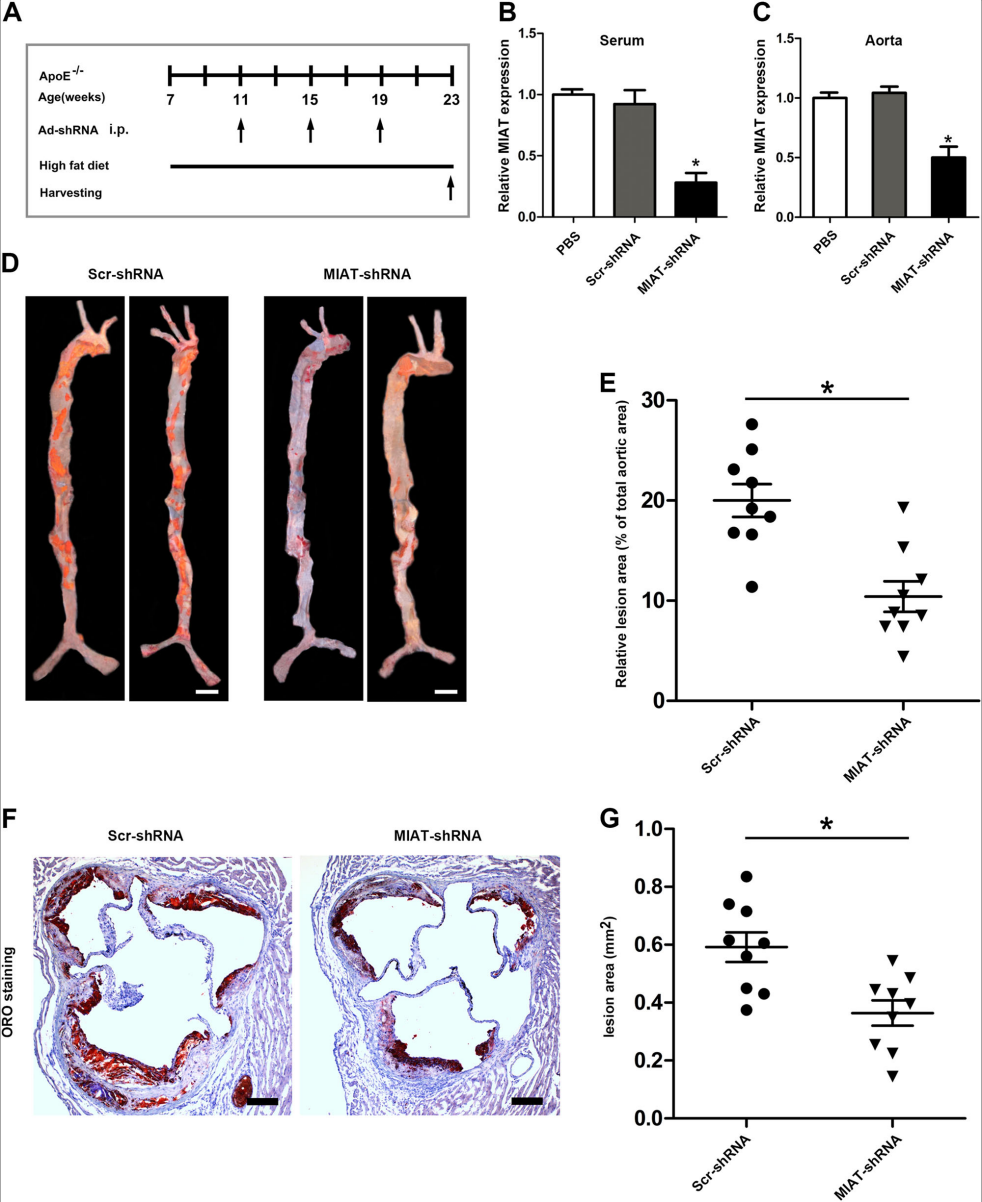
Knockdown of MIAT attenuated atherosclerosis progression in ApoE^−/−^ mice. **a** Flow charts showing the experimental protocol used in the in vivo studies. **b**, **c** qRT-PCR analysis of expression of MIAT in the serum and aorta of atherosclerotic lesions of PBS-treated, scrambled shRNA (Scr-shRNA)- treated, and MIAT shRNA-treated ApoE^−/−^ mice (*P<0.05, MIAT shRNA-treated mice vs. PBS-treated or Scr shRNA-treated mice, *n* = 6/group). **d**, **e** ORO analysis of the percentage of lesion area to total aortic area in the Scr shRNA-treated and MIAT shRNA-treated ApoE^−/−^ mice (*P<0.05, MIAT shRNA-treated mice vs. Scr shRNA-treated mice, *n* = 9/group). (Scale bar = 2 mm). **f**, **g** ORO analysis of the relative lesion area of aortic root area in the Scr shRNA-treated and MIAT shRNA-treated ApoE^−/−^ mice (*P<0.05, MIAT shRNA-treated mice vs. Scr shRNA-treated mice, *n* = 9/group). (Scale bar = 200 μm)

To explore the function and therapeutic potential of MIAT in vivo, seven-week-old ApoE^−/−^ mice were fed the HFD for 4 weeks and then treated with PBS, viral scrambled small hairpin RNA (Scr shRNA), or MIAT shRNA and continued to be fed the HFD for 12 weeks (Fig. 3a). First, we determined the efficacy of MIAT inhibition by measuring the expression of MIAT in the serum and aorta after 12 weeks of treatment. Expression levels of MIAT detected using qRT-PCR were significantly decreased in the MIAT shRNA-treated group compared with that in the Scr shRNA-treated group or PBS-treated group (Fig. 3b, c). After 12 weeks of feeding the animals with the HFD, there were no differences in body weight or levels of total cholesterol (TC), triglycerides (TG), low-density lipoproteins (LDL), or high-density lipoproteins (HDL) among the MIAT shRNA-treated group, the scrambled shRNA-treated group, or PBS-treated group (Supplementary Figure 2). Quantification of the atherosclerotic lesions by Oil-red O staining after 12 weeks of HFD showed no significant differences between the Scr shRNA-treated group and the PBS-treated group (Supplementary Figure 3). However, we found a marked reduction of plaque formation in the aorta and aortic sinus in the MIAT shRNA-treated group compared with that of the scrambled RNA-treated group (Fig. 3d–g). These results suggested that MIAT knockdown could reduce the atherosclerotic burden in vivo independent of the plasma lipid profile and weight.

### Knockdown of MIAT promoted atherosclerotic plaque stability in ApoE^−/−^ mice

**Fig. 4:**
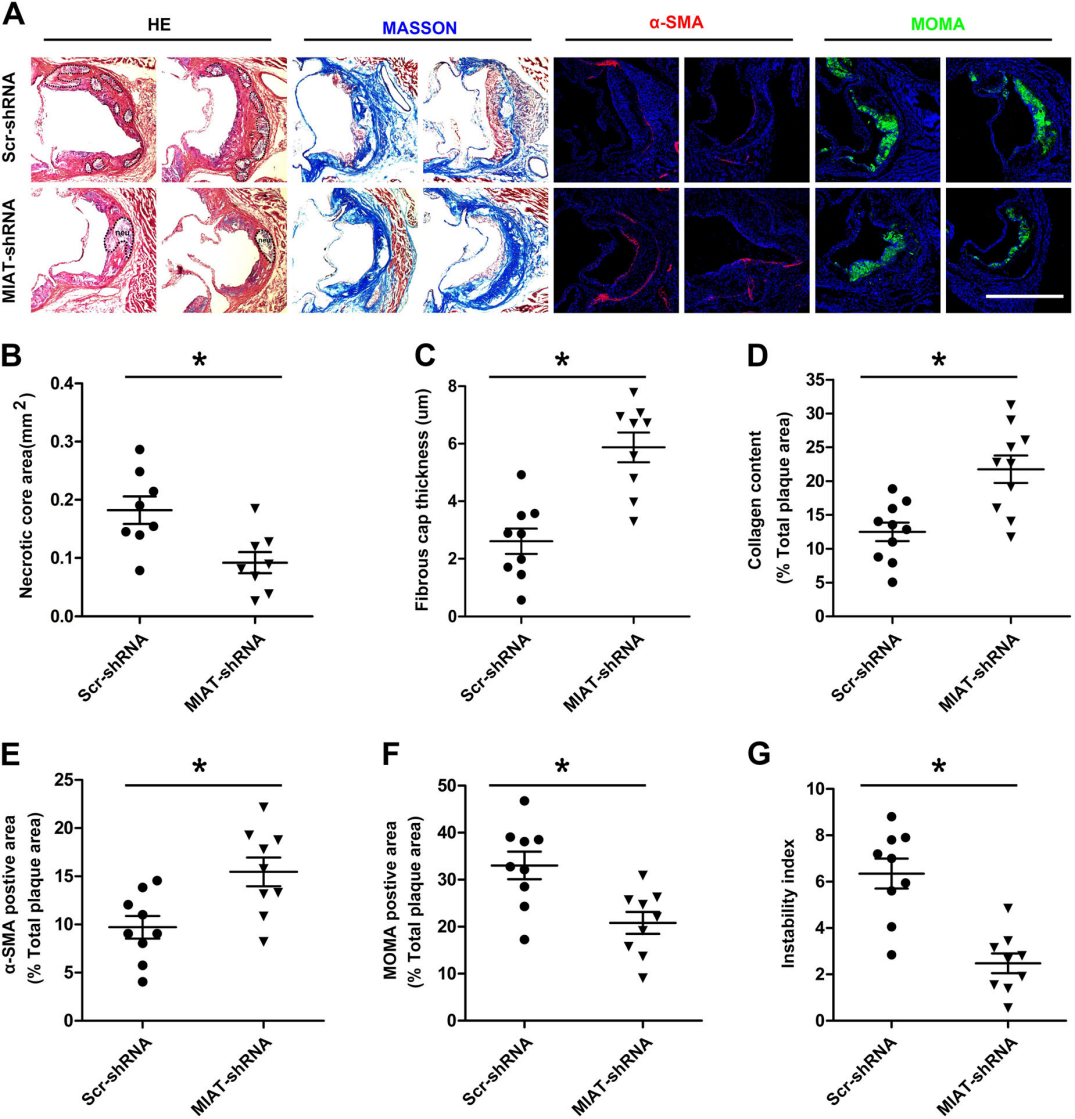
Knockdown of MIAT promoted atherosclerotic plaque stability in ApoE^−/−^ mice. **a** Cross-section histological and fluorescence microscopy analysis of aorta roots in the Scr-shRNA-treated and MIAT shRNA-treated ApoE^−/−^ mice by stained with HE, MASSON, α-SMA, or MOMA-2. Dashed lines encircle the necrotic core area. nec necrotic core. (Scale bar = 200 μm). **b–g** Quantification of average necrotic core areas, fibrotic cap thickness, the ratio of the collagen content, α-SMA-positive smooth muscle cells, or MOMA-2-positive macrophages versus the aortic root plaque area, and the instability index in the Scr shRNA-treated and MIAT shRNA-treated ApoE^−/−^mice (*P<0.05, MIAT shRNA-treated mice vs. Scr shRNA-treated mice, *n* = 8–10 per group)

Atherosclerotic plaques in the MIAT shRNA-treated group contained smaller necrotic core areas compared with that of the scrambled RNA-treated group (Fig. 4a, b). Immunohistochemical analysis showed that the collagen content and the thickness of the fibrous cap were significantly increased in the atherosclerotic plaques of the MIAT shRNA-treated group, while the MOMA-2^+^ area was significantly decreased (Fig. 4a–f). In light of these changes, the plaque instability indexes in the MIAT shRNA-treated group were decreased by several magnitudes relative to the scrambled RNA-treated group (Fig. 4g). The plaque instability index was calculated according to the following formula: [(oil red O^+^ area) + (MOMA-2^+^ area)]/[(α-SMA^+^ area) + (collagen area)]^28^. Both the decrease in necrotic area and increase in fibrous cap thickness indicated that the knockdown of MIAT promoted plaque stability.

### Knockdown of MIAT improved efferocytosis of atherosclerotic plaques in ApoE^−/−^ mice

**Fig. 5:**
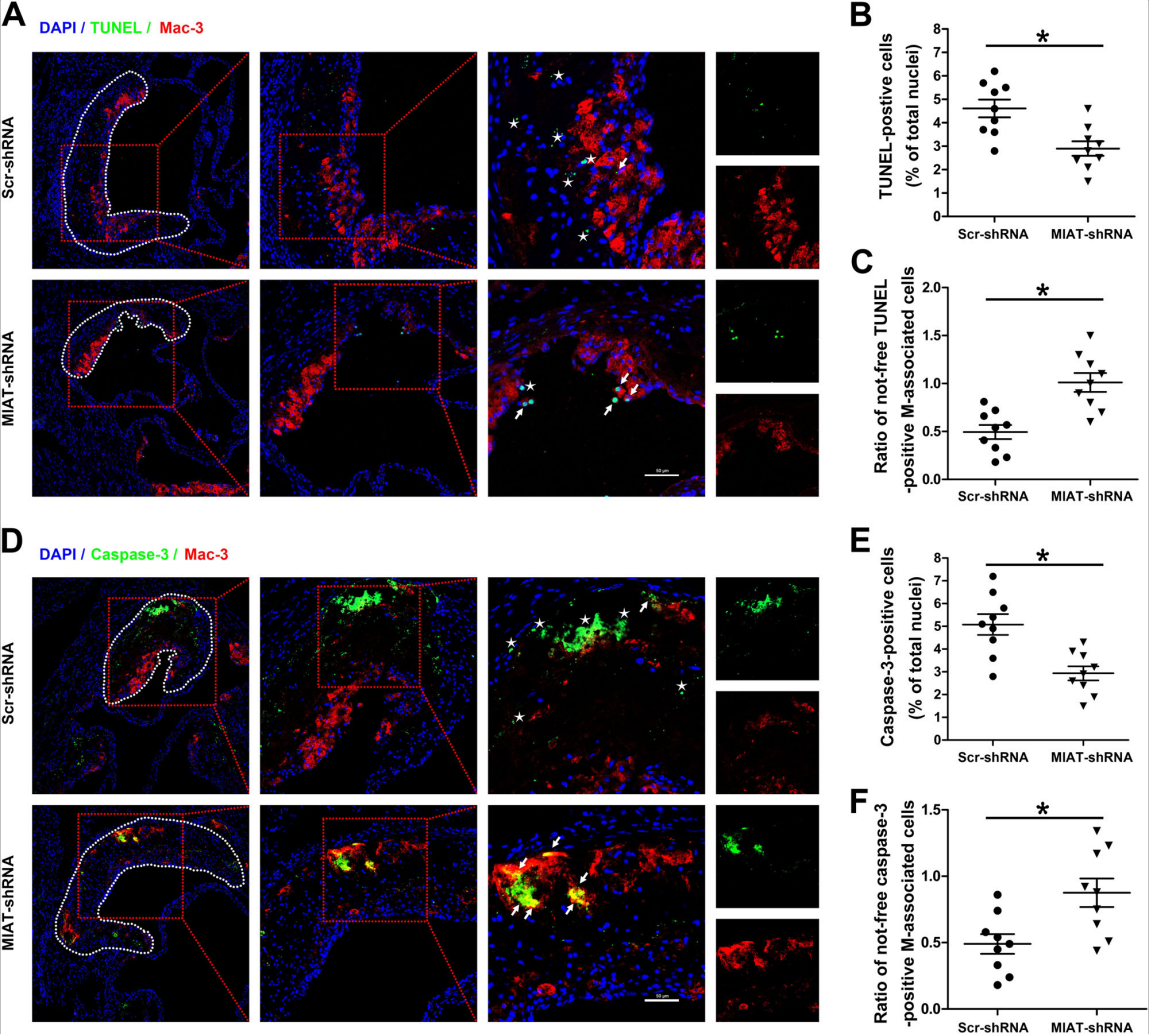
Knockdown of MIAT improved efferocytosis of atherosclerotic plaques in ApoE^−/−^ mice. **a**, **d** Representative images of aortic root sections in which apoptotic cells were labeled by TUNEL or cleaved caspase-3 (green), macrophages by Mac-3 (red), and nuclei by Hoechst (blue) (scale bar = 50 μm). **b**, **e** Data are presented as the percentage of positive cells versus the total number of nuclei (*P<0.05, MIAT shRNA-treated mice vs. Scr-shRNA treated mice, *n* = 9/group). **c**, **f** Each TUNEL or cleaved caspase-3-positive cell was determined to be either associated with a macrophage (‘not-free’ apoptotic bodies) or not associated with a macrophage (‘free’ apoptotic bodies). The data are presented as the ratio of not-free apoptotic bodies to free apoptotic cells (stars indicate ‘free’ apoptotic bodies, arrows indicate ‘not-free’ apoptotic bodies, *P<0.05, MIAT shRNA-treated mice vs. Scr shRNA-treated mice, *n* = 9/group)

A major cause of necrotic core formation is defective efferocytosis in advanced atherosclerotic plaques causing secondary necrosis of apoptosis cell-induced inflammation^18,19^. We first evaluated intraplaque apoptotic cells by detecting TUNEL-positive nuclei (Fig. 5a, b) or caspase-3 activation (Fig. 5d, e) and observed fewer apoptotic cells in the lesions of the MIAT shRNA-treated group compared to those in the other groups. To further determine whether the reduced apoptotic cell number was associated with improved efferocytosis, we examined the number of ‘free’ apoptotic bodies (no co-staining with macrophages) and ‘not-free’ apoptotic bodies (co-staining with macrophage marker Mac-3), which indicated an efferocytic event (Fig. 5a–d). Data are presented as the ratio of not-free/free apoptotic bodies, with higher values representing higher levels of efferocytosis. By either measurement, we determined that efferocytosis was significantly increased in the lesions of the MIAT shRNA-treated group compared with that of the scrambled RNA-treated group (Fig. 5c–f). These results suggested that improved efferocytosis from inhibiting MIAT may explain the decrease of necrotic core and the increase of plaque stability in the MIAT shRNA-treated group.

### Knockdown of MIAT promoted phagocytosis in vitro

**Fig. 6:**
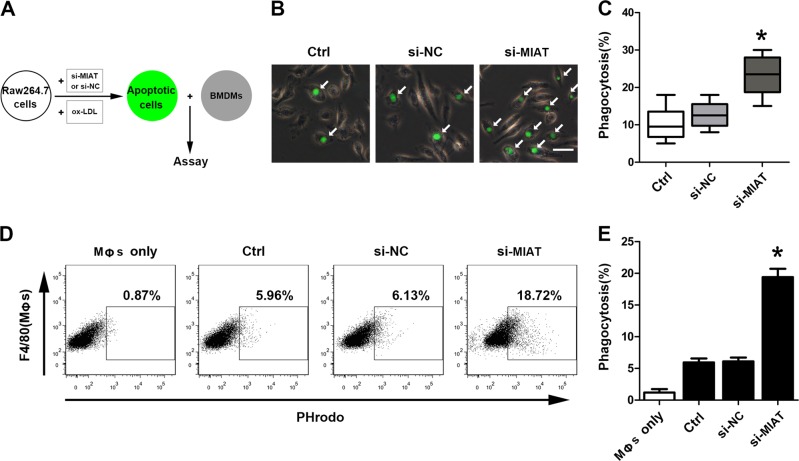
Knockdown of MIAT promoted phagocytosis in vitro. **a** Flow charts showing the phagocytosis assay protocol used in the in vitro studies. Bone-marrow-derived macrophages (BMDMs, brown) were tested for phagocytosis of apoptotic Raw264.7 cells (green) after ox-LDL treatment (150 μg/mL). **b** Raw264.7 cells transfected with or without siRNA after ox-LDL treatment (150 μg/mL) were fluorescently labeled green using CFSE and incubated with BMDM for 2 h and then examined by fluorescence microscopy. Arrows indicate BMDM-derived macrophages containing phagocytosed apoptotic Raw264.7 cells. (Scale bar = 50 μm). **c** The phagocytic index (number of target cells ingested per 100 macrophages) was determined for the indicated cell lines (*P <0.05, si-MIAT vs. Ctrl, *n* = 6/group). **d** Same as B, Raw264.7 cells transfected with or without siRNA after ox-LDL treatment (150 μg/mL) were fluorescently labeled green using pHrodo and incubated with BMDM for 2 h and then stained with APC-conjugated anti-F4/80. The stained cells were analyzed by flow cytometry. **e** The phagocytosis efficiency was determined from the percentage of F4/80^+^ cells containing pHrodo-derived green fluorescence (*P<0.05, si-MIAT vs. Ctrl, *n* = 6/group)

We next used an established in vitro phagocytosis assay to further elucidate the role of macrophage MIAT in efferocytosis (Fig. 6a). Previous studies suggested that ox-LDL may play a pre-eminent function in atherosclerotic lesion formation. Therefore, we first used ox-LDL (150 μg/mL) in vitro to mimic the stimulation of macrophage apoptosis induction in atherosclerotic plaques (Supplementary Figure 4). We designed three MIAT small interfering RNAs (siRNAs) to assure the specificity of MIAT knockdown and that MIAT knockdown did not affect apoptosis in Raw264.7 cells (Supplementary Figure 5). Based on labeling with carboxy fluorescein succinimidyl ester (CFSE), we determined that MIAT knockdown markedly increased the uptake of Raw264.7 cells transfected with si-MIAT (Fig. 6b, c). We also found that MIAT knockdown markedly increased phagocytosis compared with that of the control groups, according to a pHrodo-based assay, which identified apoptotic cells within macrophage phagolysosomes (Fig. 6d, e). Consistently, the over-expression of MIAT markedly decreased the phagocytosis of apoptotic Raw264.7 cells compared with that of the control groups (Supplementary Figure 6). These in vitro results were consistent with the in vivo data, strongly suggesting that MIAT knockdown potently induced the clearance of diseased and apoptotic macrophages that had been exposed to oxidized phospholipids to simulate the atherosclerotic environment.

### MIAT upregulated anti-phagocytic molecule CD47 expression in a transcription-independent manner

**Fig. 7:**
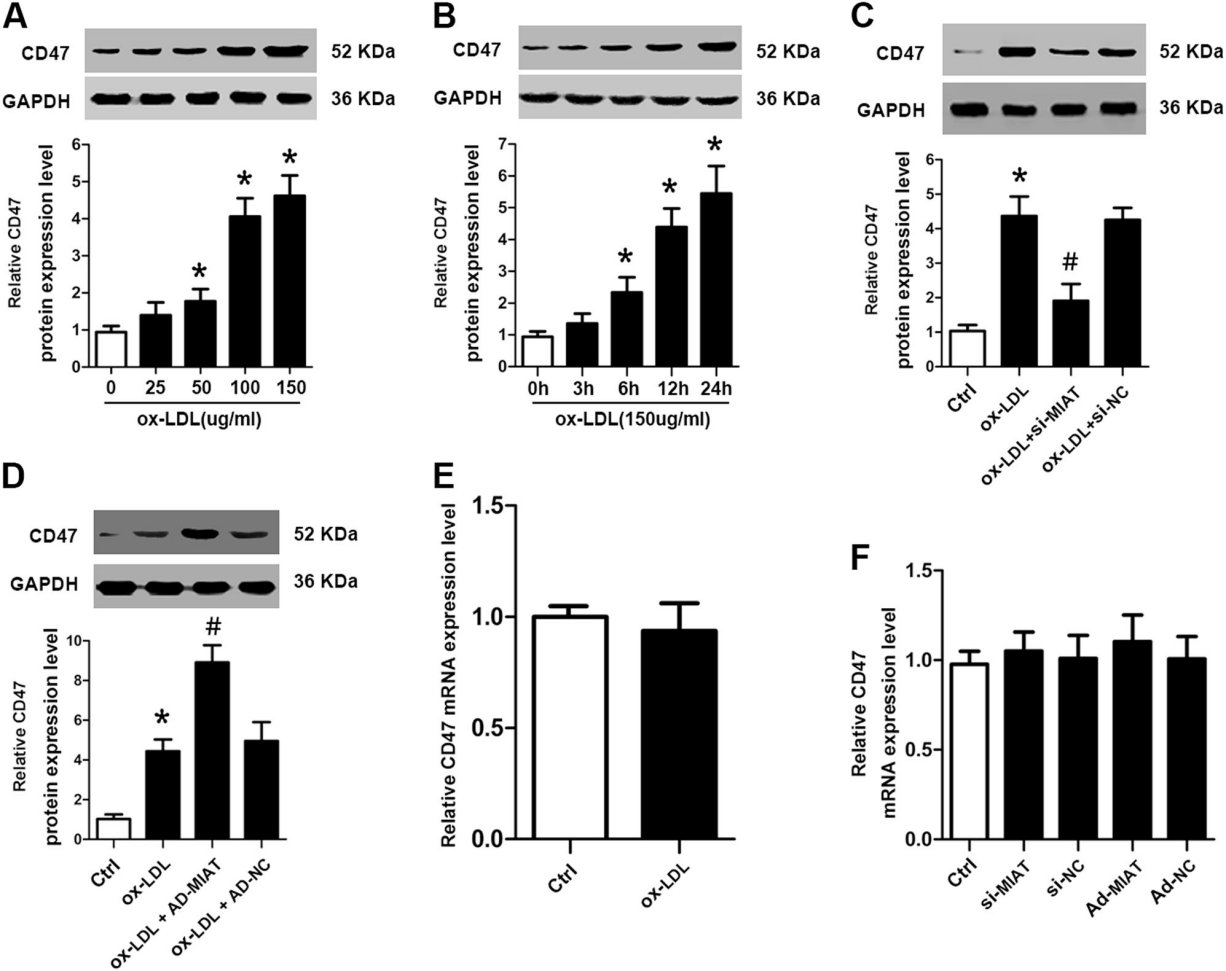
MIAT increased CD47 protein levels after ox-LDL treatment. **a**, **b** Western blot analysis of CD47 protein expression in ox-LDL-treated Raw264.7 cells at the indicated dose (0, 25, 50, 100, and 150 μg/mL) and at the indicated time (0, 3, 6, 12, and 24 h) (*P<0.05 vs. Ctrl, *n* = 6/group). **c** Western blot analysis of CD47 protein expression in ox-LDL-treated Raw264.7 cells with or without knockdown of MIAT (*P<0.05, ox-LDL vs. Ctrl, ^#^*P*<0.05, ox-LDL + si-MIAT vs. ox-LDL, *n* = 6/group). **d** Western blot analysis of CD47 protein expression in ox-LDL-treated Raw264.7 cells with or without over-expressed of MIAT (*P<0.05, ox-LDL vs. Ctrl, ^#^*P*<0.05, ox-LDL + si-MIAT vs. ox-LDL, *n* = 6/group). **e** qRT-PCR analysis of CD47 mRNA expression in Raw264.7 cells after ox-LDL treatment. **f** qRT-PCR analysis of CD47 mRNA expression in Raw264.7 cells with under-expressed or over-expressed MIAT

A recent study has shown that unengulfed macrophages express the anti-phagocytic molecule CD47 in advanced mouse and human atherosclerotic lesions^24^. We first found that the protein levels of CD47 were significantly upregulated after ox-LDL treatment in dose-dependent and time-dependent manners, which was consistent with MIAT expression after ox-LDL treatment (Fig. 7a, b). To determine whether CD47 might be directly responsible for the role of MIAT in defective efferocytosis, we performed in vitro gain-of-function and loss-of-function experiments in macrophages to investigate the effect of MIAT on CD47. Knockdown of MIAT using siRNA transfection decreased the protein levels of CD47 with ox-LDL treatment compared with that of the control group (Fig. 7c). Conversely, the over-expression of MIAT by adenovirus infection efficiently increased ox-LDL-induced CD47 expression (Fig. 7d). We next investigated whether MIAT upregulated CD47 expression in a transcription-dependent manner. Interestingly, ox-LDL treatment did not affect the mRNA levels of CD47 in macrophages (Fig. 7e). Furthmore, downregulation and upregulation of MIAT did not change the CD47 mRNA levels (Fig. 7f). These data suggested that MIAT regulated CD47 expression in a transcription-independent manner.

### MIAT regulated CD47 expression by acting as a sponge of miR-149-5p

**Fig. 8:**
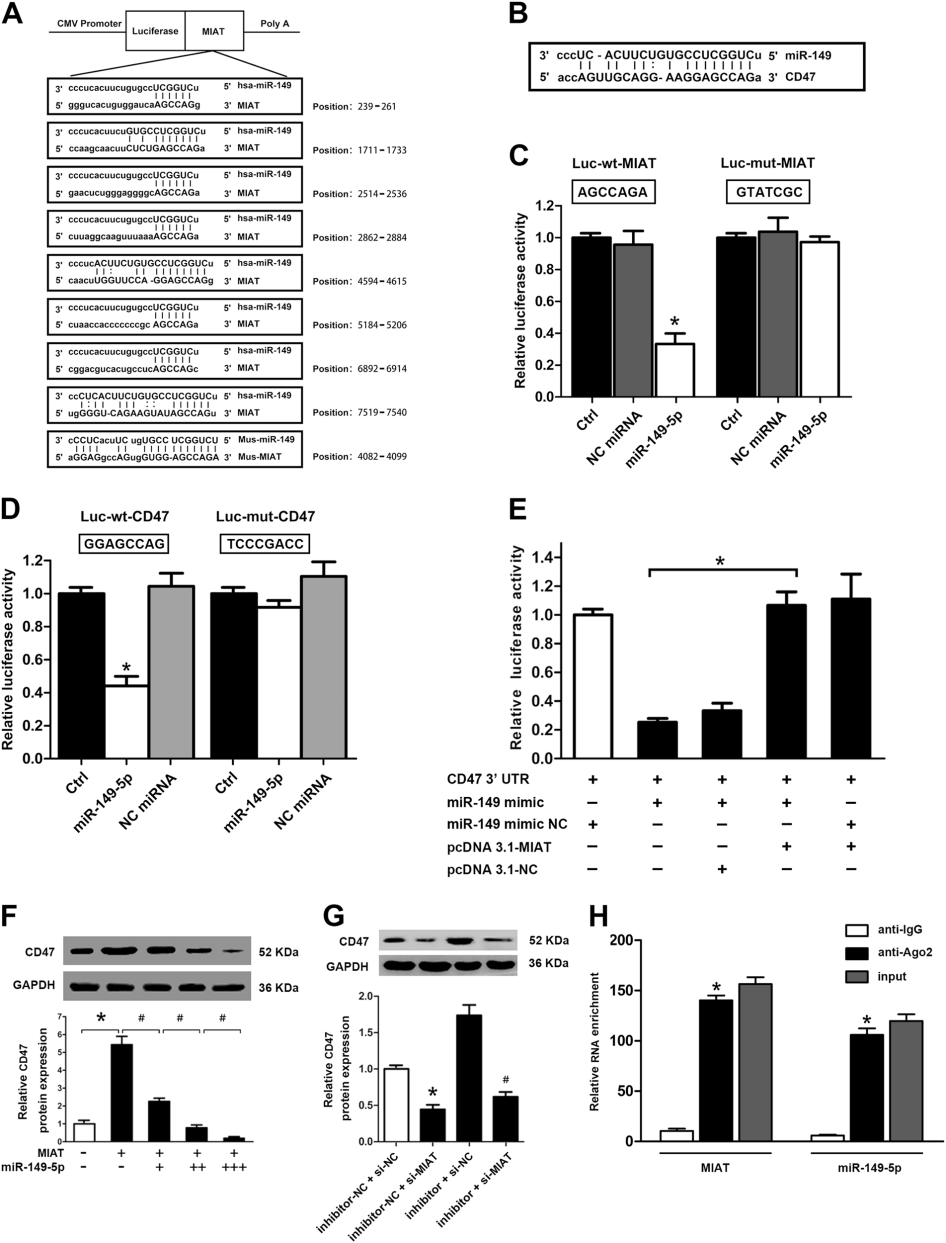
MIAT regulated CD47 expression by acting as a sponge of miR-149-5p. **a** StarBase prediction indicated that the MIAT sequence contained eight putative miR-149-5p sites in humans and one putative miR-149-5p site in mice. **b** StarBase analysis indicated that CD47 was predicted to be a target gene of miR-149-5p. The positions of miR-149-5p binding sites on CD47 are shown. **c** The cDNA of MIAT was cloned downstream of the luciferase gene (Luc-MIAT-Wt) and transfected into HEK293 cells with miR-149-5p mimic or negative control (NC). To avoid unspecific binding, the miR-149-5p binding sites in MIAT were mutated to generate Luc-MIAT-Mut. Luciferase activity was detected 48 h after transfection (*P<0.05 vs. NC, *n* = 6/group). **d** Same as **c**, miR-149-5p mimics or NC were co-transfected with Luc-CD47-WT or MUT into HEK293 cells. Luciferase activity was detected 48 h after transfection (*P<0.05 vs. NC, *n* = 6/group). **e** Luc-CD47-WT and miR-149-5p were co-transfected into HEK293 cells with MIAT plasmid or the vector to verify the competing endogenous RNA activity of MIAT. Luciferase activity was detected 48 h after transfection (*P<0.05 vs. NC, *n* = 6/group). **f** Raw264.7 cells were transfected with different combinations of MIAT and miR-149-5p mimic and the cells were treated with ox-LDL. Western blot analysis was conducted to detect CD47 expression. The plus sign (+) corresponds to Ad-MIAT or 25 ng of miR-149-5p mimic. The double plus signs (++) corresponds to 50 ng of miR-149-5p mimic. The triple plus signs (+++) corresponds to 100 ng of miR-149-5p mimic (*P<0.05, Ad-MIAT vs. Ctrl, ^#^*P*<0.05, Ad-MIAT + miR-149-5p mimic vs. Ad-MIAT, *n* = 6/group). **g** Raw264.7 cells with or without si-MIAT were transfected with miR-149-5p inhibitor or inhibitor control and the cells were treated with ox-LDL. Western blot analysis was conducted to detect CD47 expression (*P<0.05, si-MIAT + inhibitor-NC vs. si-NC + inhibitor-NC, ^#^*P*<0.05, si-MIAT + miR-149-5p inhibitor vs. si-MIAT + inhibitor NC, *n* = 6/group). **h** RNA-binding protein immunoprecipitation (RIP) assays were performed using input from cell lysates, normal mouse IgG, or anti-Ago2. Relative expression levels of MIAT and miR-149-5p in Raw264.7 cells were detected by qRT-PCR analysis

Numerous studies have shown that cytoplasmic lncRNAs can serve as natural miRNA sponges, which interferes with miRNA at the post-transcriptional level and decreases binding of endogenous miRNAs to target genes^5,29^. We searched an online bioinformatics database (http://34.236.212.39/microrna/getGeneForm.do) for the potential miRNA recognition elements on MIAT. We found that the MIAT sequence contained eight putative miR-149-5p sites in humans, and one putative miR-149-5p site in mice (Fig. 8a). Interestingly, using miRbase CD47 was also predicted to be a target gene of miR-149-5p (Fig. 8b). The sequences of MIAT or CD47 3′UTR along with the miR-149-5p binding site and the mutations MIAT mut or CD47 3′UTR mut were inserted downstream of the luciferase gene in the reporter plasmid for use in luciferase assays. The luciferase assay revealed that miR-149-5p transfection could reduce MIAT expression in the wild-type group compared with that in the negative control group and this inhibition was reversed when the binding site was mutated, suggesting the same sequence-specific binding of miR-149-5p to MIAT or CD47 3′UTR (Fig. 8c, d).

To further determine the relationship among MIAT, miR-149-5p, and CD47, the CD47 3′UTR was co-transfected with miR-149-5p and the MIAT plasmid (pcDNA-MIAT) and analyzed by using luciferase assays. The results of the luciferase activity showed that MIAT could counteract the inhibitory effects of miR-149-5p on CD47 expression (Fig. 8e). Consistent with these findings, the over-expression of MIAT in Raw264.7 cells increased the protein levels of CD47 with this effect being counteracted by the over-expression of miR-149-5p (Fig. 8f). Knockdown of MIAT in Raw264.7 cells decreased the protein levels of CD47, which was also reversed by the down-regulation of miR-149-5p (Fig. 8g). These data suggested that MIAT modulated the expression of CD47 by interaction with miR-149-5p. Finally, to further confirm that MIAT and miR-149-5p were associated through a sponge, RNA immunoprecipitation was performed on Raw264.7 cell extracts using an antibody specific for Ago2, a core component of the RNA-induced silencing complex (RISC). We found that higher MIAT and miR-149-5p RNA levels were detected in the Ago2-containing miRNA ribonucleoprotein complexes (Fig. 8h). Therefore, these results indicated that MIAT acted as a miR-149-5p sponge and elevated CD47 expression.

## Discussion

**Fig. 9:**
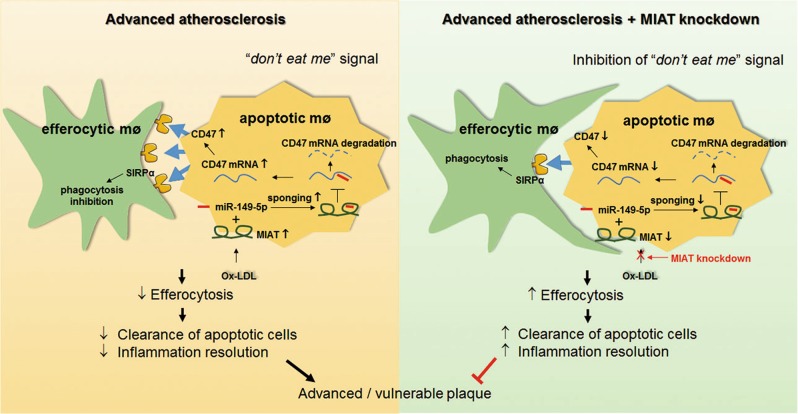
Schematic representation to show the roles of lncRNA MIAT in defective efferocytosis and plaque vulnerability. lncRNA MIAT functions as a decoy by competitively binding miR-149-5p, preventing its interaction with CD47, and increasing ox-LDL induced CD47 upregulation, promoting defective efferocytosis and plaque vulnerability

Recent studies have reported that lncRNA MIAT has critical functions in many cancers and microvascular dysfunction^11,30^, but little is known about its roles in the progression and instability of atherosclerosis. In the current study, we first found that MIAT expression levels in the serum were elevated in symptomatic patients with vulnerable plaques and HFD-fed ApoE^−/−^ mice with advanced atherosclerosis. Expression of MIAT was primarily located in macrophages in the lesions and was also significantly increased after ox-LDL treatment. Furthermore, we determined that systemically delivered viral MIAT shRNA significantly reduced atherosclerosis progression in vivo and promoted plaque stability by enhancing the clearance of apoptotic cells by lesion macrophages in ApoE^−/−^ mice. Specifically, our gain-of-function and loss-of-function experiments demonstrated that MIAT regulated miR-149-5p/CD47 axis to improve efferocytosis in vitro (summarized in Fig. 9). These findings support the rationale that MIAT acts as a novel biomarker and may serve as a therapeutic target for managing the progression and vulnerability of atherosclerotic disease.

LncRNAs are critical regulators of various biological functions and altered expression is closely associated with the development and progression of various diseases^5,7,29^. Recent studies have shown that several lncRNAs such as lncRNA MeXis, lncRNA LeXis, and lncRNA SMILR are involved in macrophage cholesterol efflux, cholesterol metabolism, and SMCs proliferation, which are important in the process of atherosclerotic disease^31–33^. However, few studies have demonstrated regulatory functions of lncRNAs in atherosclerotic plaque vulnerability. Plaque vulnerability, which is responsible for plaque rupturing, is more highly correlated with plaque composition than with plaque size^3,19,34^. In the clinic, atherosclerotic plaque rupture is the most important mechanism of acute ischemic stroke^35,36^. The average annual rate of ipsilateral stroke in patients with asymptomatic carotid stenosis is ~0.5–1% per year and patients with low to moderate carotid stenosis also suffer strokes. Therefore, it is of great value to identify potential biomarkers of vulnerable plaques influencing the local disease process, which may provide reasonable solutions for these individuals.

In our current study, by comparing locally sampled serum of patients who were either asymptomatic or symptomatic (had recently suffered a transient ischemic attack or stroke), we identified MIAT as an important lncRNA being synthetized and released into the local cellular milieu. Consistent with these findings, recent studies have shown that MIAT is specifically up-regulated in the serum or plasma of diabetic cardiomyopathy patients and cataract patients^37,38^. In addition, a study reported that MIAT of peripheral blood leukocytes might be a potential diagnostic and prognostic indicator in ischemic stroke^15^. Furthermore, using RNA-FISH analysis we confirmed that MIAT was predominantly localized in the macrophages of advanced mouse atherosclerotic plaques. These results suggested that elevated MIAT positively correlated with atherosclerosis progression and instability. Due to the limitation in the number of clinical samples in our current study, a larger cohort study with follow-up information is needed, which would provide reliable data to fully determine whether MIAT is associated with prognosis or can serve as a new biomarker for vulnerable plaques.

Recent work has shown that MIAT knockdown alleviates diabetic-induced retinal neovascularization, vascular leakage, and inflammation in vivo^11,37,39^. To study the function of MIAT and examine the therapeutic potential in atherosclerosis, we knocked-down MIAT in vivo using systemically delivered viral MIAT shRNA in ApoE^−/−^ mice. Interestingly, MIAT knockdown significantly enhanced clearance of apoptotic cells by macrophages both in vivo and in vitro, which may explain the decrease of necrotic core and the increase of plaque stability. The anti-phagocytic molecule CD47 has been closely associated with the clearance of apoptotic cells and the formation of necrotic cores in plaque lesions^24^. In the present study, by performing in vitro gain-of-function and loss-of-function experiments in macrophages we determined that MIAT could positively regulate CD47 expression in a transcription-independent manner. Thus, it can be speculated from these results that CD47 may be a downstream target of MIAT in efferocytosis.

LncRNAs have been reported to be located in both the cytoplasm and nucleus of cells and subcellular localization patterns of lncRNAs provide new insight into their function and boost the hypotheses for potential molecular mechanisms^5,7,40^. First, by performing RNA FISH assays, we found that MIAT was localized in the cytoplasm of macrophages and levels in the cytoplasm and nucleus increased after ox-LDL treatment. The results indicated that MIAT might participate in post-transcriptional regulations in the cytoplasm^41,42^. Recently, a novel mechanism of post-transcriptional regulation by lncRNAs has been identified in which it functions as a natural miRNA sponge, interferes with miRNA pathways, and regulates the de-repression of miRNA targets^41,43^. Second, we predicted the interaction between MIAT and miR-149-5p using an online bioinformatics database and found that MIAT contains a target site of miR-149-5p. In addition, CD47 was predicted to be a target gene of miR-149-5p. Third, the over-expression of MIAT increased the level of CD47 and this effect was counteracted by the over-expression of miR-149-5p. Knockdown of MIAT decreased the level of CD47, which also was reversed by the down-regulation of miR-149-5p. Finally, we found that MIAT and miR-149-5p were in the same RISC complex. Therefore, we proposed that MIAT acted as a ceRNA to regulate CD47 expression by sponging miR-149-5p in macrophages, which may be one of the mechanisms by which MIAT acts as a critical regulator in intraplaque efferocytosis and atherosclerotic plaque vulnerability.

The emergence of atherosclerosis-related lncRNAs as regulators of gene expression has greatly altered our understanding of the pathological mechanisms of atherosclerosis. In summary, we present evidence that lncRNA MIAT is a key regulatory factor for plaque vulnerability. These data also demonstrated how MIAT inhibited efferocytosis by targeting miR-149-5p/CD47 axis. The modulation of MIAT may provide an intriguing approach for prevention of atherosclerosis and tackling its treatment.

## Materials and methods

### Ethics statement

All the in vivo experiments complied with the Guide for Care and Use of Laboratory Animals published by the United States National Institutes of Health (NIH Publication, 8th Edition, 2011) and were approved by the Animal Care Committee of Tongji Medical College, Huazhong University of Science and Technology. The research protocol was approved by the ethical committee of Union Hospital, Huazhong University of Science and Technology, Wuhan, China. Written informed consent was obtained from each of the patients or the appropriate agents.

### Study population

The study population consisted of 38 patients with carotid atherosclerosis and 20 control individuals. The participants were consecutively recruited from the Neurology Department and Health Examination Center between April 2016 and December 2017 at Union Hospital, Huazhong University of Science and Technology, Wuhan, China. The patients with ischemic stroke in the internal carotid artery underwent carotid vessel wall and brain magnetic resonance (MR) examinations within one week of symptoms onset. The exclusion criteria were as follows: (1) patients with evidence of cardioembolic stroke, (2) patients with hemorrhagic stroke, (3) history of radiation therapy of the neck, (4) other etiologies such as vasculitis or moyamoya disease, and (5) contraindications to magnetic resonance imaging (MRI).

### Magnetic resonance imaging and analysis

For carotid vessel wall imaging, a previously published multi-contrast protocol was used for plaque detection that included T1-weighted, T2-weighted, 3D sampling perfection with application optimized contrasts using different flip angle evolutions (3D-SPACE), and post-contrast T1 and T2-weighted sequences^44,45^. Each lesion was classified according to the modified American Heart Association (AHA) classification for MRI^46,47^. Briefly, Type I–III were classified as stable plaques while types IV–V and VI were classified as unstable plaques. Each axial image was reviewed by two reviewers with consensus being reached. The reviewers were blinded to all clinical information.

### Plasma collection and storage

Peripheral blood plasma from the patient group and control group were collected in ethylenediaminetetraacetic acid (EDTA) tubes and processed within 2 h by centrifugation at 1000 × *g* at 4 °C for 10 min. The plasma was gently transferred to RNase/DNase-free 2-mL EP tubes (Axygen, Union City, USA) and cryopreserved at −80 °C until use.

### Atherosclerosis animal model protocol

A total of 100 seven-week-old male ApoE^−/−^ mice were purchased from Vital River Laboratory Animal Technology (Beijing, China). Thirty of the ApoE^−/−^ mice were randomly divided into two groups, a normal chow diet group (NCD, *n* = 15) and a high-fat diet group (HFD, *n* = 15). The remaining 70 ApoE^−/−^ mice were fed a high-fat diet and randomly divided into three groups, a PBS group (*n* = 10), Scr-shRNA group (*n* = 30), and MIAT-shRNA group (*n* = 30). The 7-week-old male ApoE^−/−^ mice were fed a high-fat diet containing 21.2% fat (1.5% cholesterol) and 16.7% protein for 16 weeks and the shRNA or PBS injections were then started at week 4 after initiation of the high-fat diet. The mice were treated intraperitoneally with shRNA adenoviral vector at a dose of 50 μl/g (virus titer: 1.0 × 10^13^ viral genomes/mL) or PBS. The Scr shRNA-treated mice were used as a control group. The body weight of the mice was measured throughout the experimental period.

### Cell culture

Raw264.7 cells and human embryonic kidney (HEK) 293 T cells were purchased from the Cell Resource Center of the Shanghai Institute for Biological Sciences (Shanghai, China). Both cell types were cultured in Dulbecco’s modified Eagle’s medium (DMEM; HyClone, USA) supplemented with 10% fetal bovine serum (FBS; Gibco, USA) and 1% penicillin/streptomycin (P/S; Thermo Fisher Scientific, Rockford, IL, USA) and were incubated in a humidified incubator at 37 °C with 5% CO_2_.

### RNA interference and virus infection

RNA interference was performed as previously described^48^. Negative control siRNA, and miR-149-5p miRNA mimics and inhibitors were designed and synthesized by Sangon Biotech (Shanghai, China). The adenovirus over-expressing lncRNA-MIAT was constructed by Shanghai R&S Biotechnology Company (Shanghai, China).

### RNA extraction and quantitative real-time PCR

Total RNA was isolated using Trizol (Invitrogen, USA). Real-time qRT-PCR was performed using a thermocycler (Bio-Rad, Hercules, CA, USA) according to the manufacturer’s recommendations. The primers for CD47 were 5′-CATGGCCCTCTTCTGATTTC-3′ (forward) and 5′-GGAGGTTGTATAGTCTTCTGATTGG-3′ (reverse). The primers for LncRNA-MIAT were 5′-ATCCTCGAGACAAAGAGCCCTCTGCACTAG-3′ (forward) and 5′-

ATCGGATCCGAGCAAATGGAGACAAAGGAC-3′ (reverse). The forward primer sequence for miR-149-5p was 5′-TCTGGCTCCGTGTCTTCACTCCC-3′. U6 and β-actin mRNA were used as housekeeping reference genes. The relative quantification of gene expression was performed using the comparative Ct method.

### Luciferase reporter assay

The assays were performed as previously described^48^. In brief, for the luciferase reporter assay, the 3′UTR of MIAT or CD47 was amplified by PCR and inserted downstream of the firefly luciferase reporter gene in the reporter vector (RiboBio, China). Point mutations of the miR-149-5p targeting sites in the MIAT or CD47 3′UTR were generated. Luciferase activity was detected using the Dual-Luciferase Reporter Assay System (Promega, Madison, WI, USA).

### RNA-binding protein immunoprecipitation (RIP) assay

RIP assays were performed using the EZ-Magna RIP Kit (Millipore, USA). Briefly, cells were collected and lysed in complete RIP lysis buffer. The cell extracts were then incubated with RIP buffer containing magnetic beads conjugated to anti-Ago2 antibody (Millipore, USA). Samples were incubated with proteinase K with shaking to digest the proteins and the immunoprecipitated RNA was isolated. MIAT and miR-149-5p levels in the precipitates were determined by qRT-PCR analysis.

### In vitro phagocytosis assays

For the pHrodo and flow cytometry-based assays bone-marrow-derived macrophages (BMDMs) were prepared for phagocytosis^49,50^. The BMDMs (5 × 10^4^) were seeded overnight in 24-well culture plates. Raw264.7 cells were induced as apoptotic cells using ox-LDL (150 μg/mL) for 24 h and then labeled with 100 ng mL^−1^ of pHrodo Green AM Intracellular pH Indicator (Thermo Fisher Scientific, USA) according to the manufacturer’s instructions. After incubating the BMDMs in serum-free medium for 2 h, 2×10^5^ pHrodo-labeled Raw264.7 cells were added to the BMDMs. After incubation for 2 h at 37 °C, the BMDMs were washed extensively, then stained with alkaline phosphatase-conjugated (APC) anti-F4/80 antibody and were analyzed by flow cytometry. The pHrodo dyes do not fluoresce under neutral pH but become fluorescent in acidic environments, such as phagolysosome. The efficiency of phagocytosis was determined from the percentage of F4/80^+^ cells containing pHrodo-derived green fluorescence. For the microscopy-based assay, apoptotic Raw264.7 cells were used for the preparation and incubation of BMDMs as detailed for the flow cytometry-based assay^50,51^. Meanwhile, the apoptotic Raw264.7 cells were labeled with 2.5 μM carboxyfluorescein succinimidyl ester (CFSE) using a CFSE Cell Proliferation Kit (C34554; Life Technologies, Burlington, Ontario, Canada). After 2 h incubation at 37 °C, the BMDMs were extensively washed and imaged using an inverted microscope (Carl Zeiss Axiovert S100 TV). The number of BMDMs containing CFSE+ apoptosis cells per 100 BMDMs was calculated and the phagocytosis efficiency accordingly evaluated.

### Biochemical parameters

As previously described^28,48^, total cholesterol (TC), triglycerides (TG), low-density lipoprotein cholesterol (LDL-c), and high-density lipoprotein cholesterol (HDL-c) were enzymatically measured (Wako Chemicals, USA) following the manufacturer’s protocols.

### Histology and immunostaining

Serial sections (6 μm thick) of the aortic root (3–5 sections per mouse) were stained with hematoxylin and eosin (HE), Oil Red O (ORO), or Masson’s trichrome (MASSON) and the microscopic images were then collected. Quantitative immunostaining was performed using primary antibodies for capase-3 (1:200, Cell Signaling Technology [CST], Danvers, MA, USA), Mac-3 (1:200, Abcam, USA), MOMA-2 (1:200, Abcam, USA), or α-smooth muscle actin (α-SMA, 1:100, Abcam, USA). Fluorescently labeled secondary antibodies were used for detection. The quantification of colocalized signals and the percentage of positive area in the images were performed using NIS Elements AR Imaging Software 4.10 (Nikon) or ImageJ 1.41 software (Image Processing and Analysis in Java; National Institutes of Health, Bethesda, MD, USA).

### In situ detection of apoptotic cells

Apoptotic cells in aortic root cryo-sections were determined using an In-Situ Apoptosis Fluorescein Detection Kit (Roche, Sigma, USA) according to the manufacturer’s instructions.

### RNA fluorescent in situ hybridization

Cy3-labeled lncRNA-MIAT and DAPI-labeled U6 probes were obtained from RiboBio (Guangzhou, China). RNA-FISH assays were carried out using a fluorescent in situ hybridization kit (RiboBio, Guangzhou, China) following the manufacturer’s instructions.

### Western blot analysis

Proteins were extracted from cell lysates using RIPA buffer containing protease inhibitors. Eighty μg protein was separated by 6% sodium dodecyl sulfate–polyacrylamide gel electrophoresis (SDS-PAGE) and transferred to polyvinylidene difluoride (PVDF) membrane. The membranes were incubated overnight with antibodies to CD47 (1:400, Abcam, USA) and capase-3 (1:500, CST, USA) followed by incubation with the corresponding secondary antibody. The bands were visualized using the enhanced chemiluminescence method. Protein expression was quantified and normalized to GAPDH (1:1000, Proteintech, China).

### Statistics

The data represent the mean ± standard error of the mean (SEM). A Kolmogorov–Smirnov normality test was performed to determine whether the data showed normal distribution. Comparisons between the controls and treatment groups were performed using either a Student’s *t*-test or one-way ANOVA. The statistical significance is shown as described in the figure legends. The Mann–Whitney *U* test or Student’s *t*-test was used for continuous variables between patients and controls. Statistical significance was defined as *P* < 0.05 for all tests. Statistical analyses were performed using the Statistical Package for the Social Sciences (SPSS) version 17.0 software (SPSS Inc., Chicago, IL, USA) and GraphPad Prism 6.

## Supplementary information

Supplementary figure 1

Supplementary figure 2

Supplementary figure 3

Supplementary figure 4

Supplementary figure 5

Supplementary figure 6

Supplementary table 1

supplemental figure legends
