# Supplementary figures and images for: LncRNA MIAT sponges miR-149-5p to inhibit efferocytosis in advanced atherosclerosis through CD47 upregulation
Source: Cell Death Dis. 2019 Feb 12;10(2):138. doi: 10.1038/s41419-019-1409-4 (PMC6372637; doi:10.1038/s41419-019-1409-4)

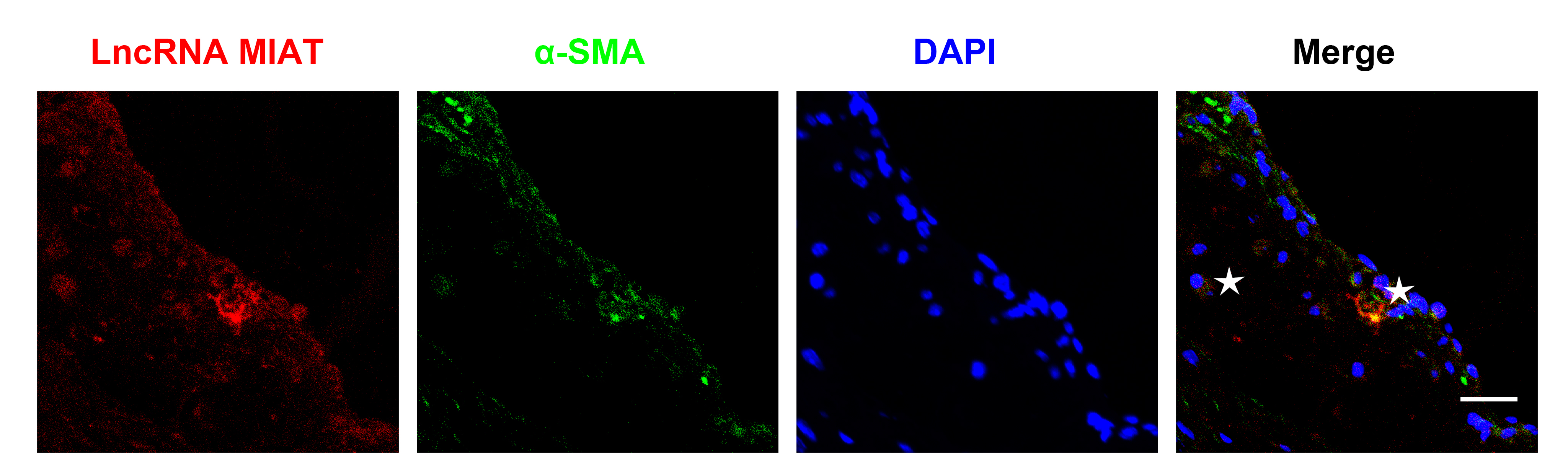

Supplement: Supplementary file 1 — Supplementary figure 1 [file 41419_2019_1409_MOESM1_ESM.jpg]

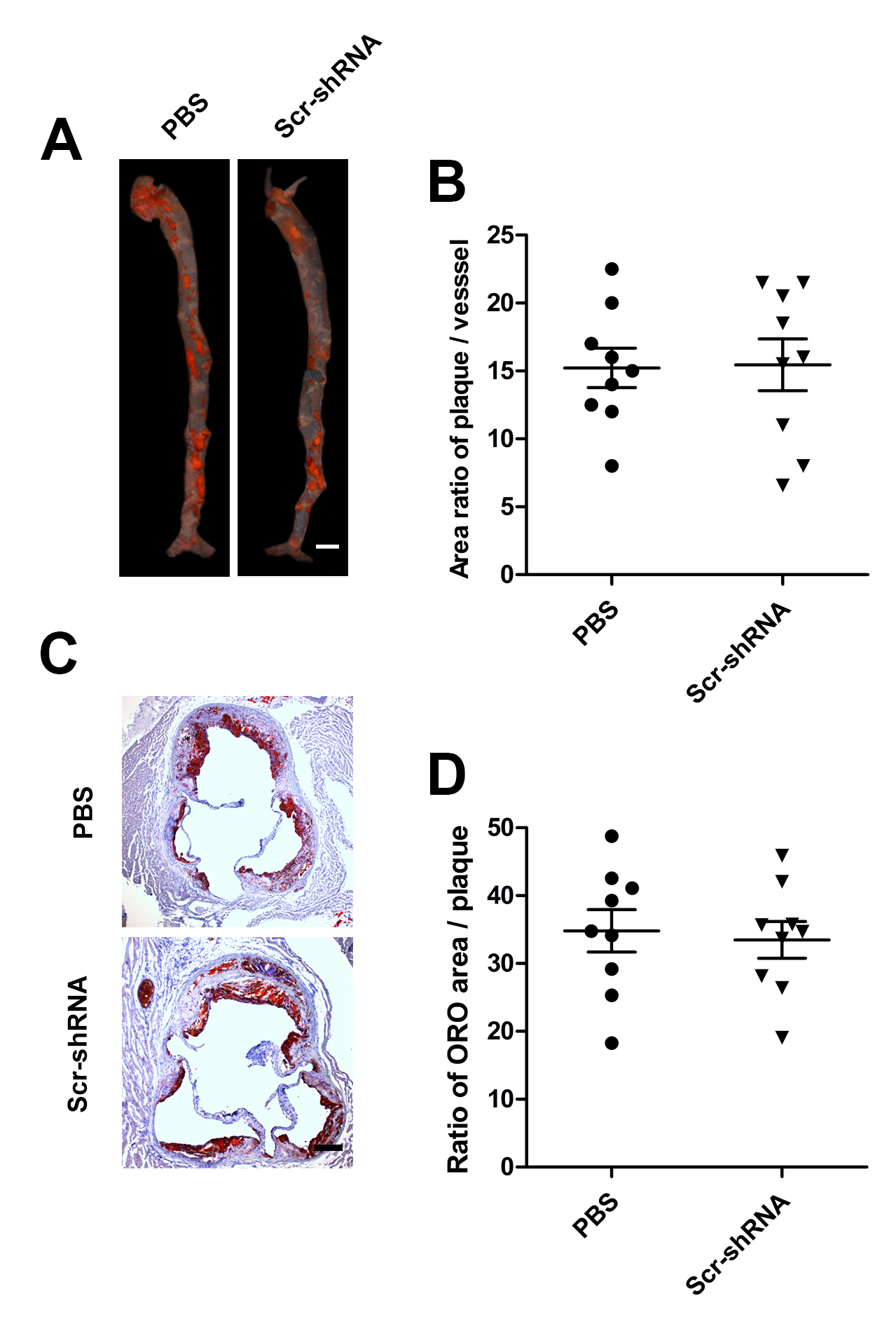

Supplement: Supplementary file 3 — Supplementary figure 3 [file 41419_2019_1409_MOESM3_ESM.jpg]

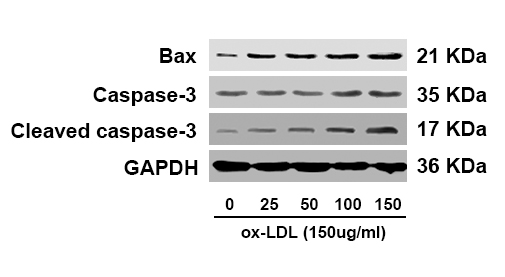

Supplement: Supplementary file 4 — Supplementary figure 4 [file 41419_2019_1409_MOESM4_ESM.jpg]

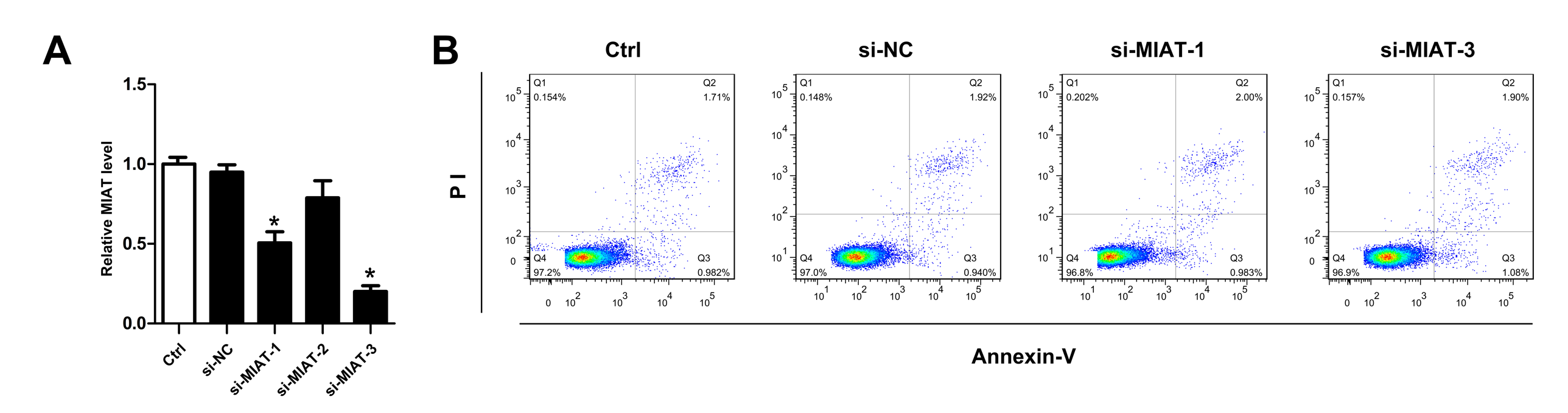

Supplement: Supplementary file 5 — Supplementary figure 5 [file 41419_2019_1409_MOESM5_ESM.jpg]

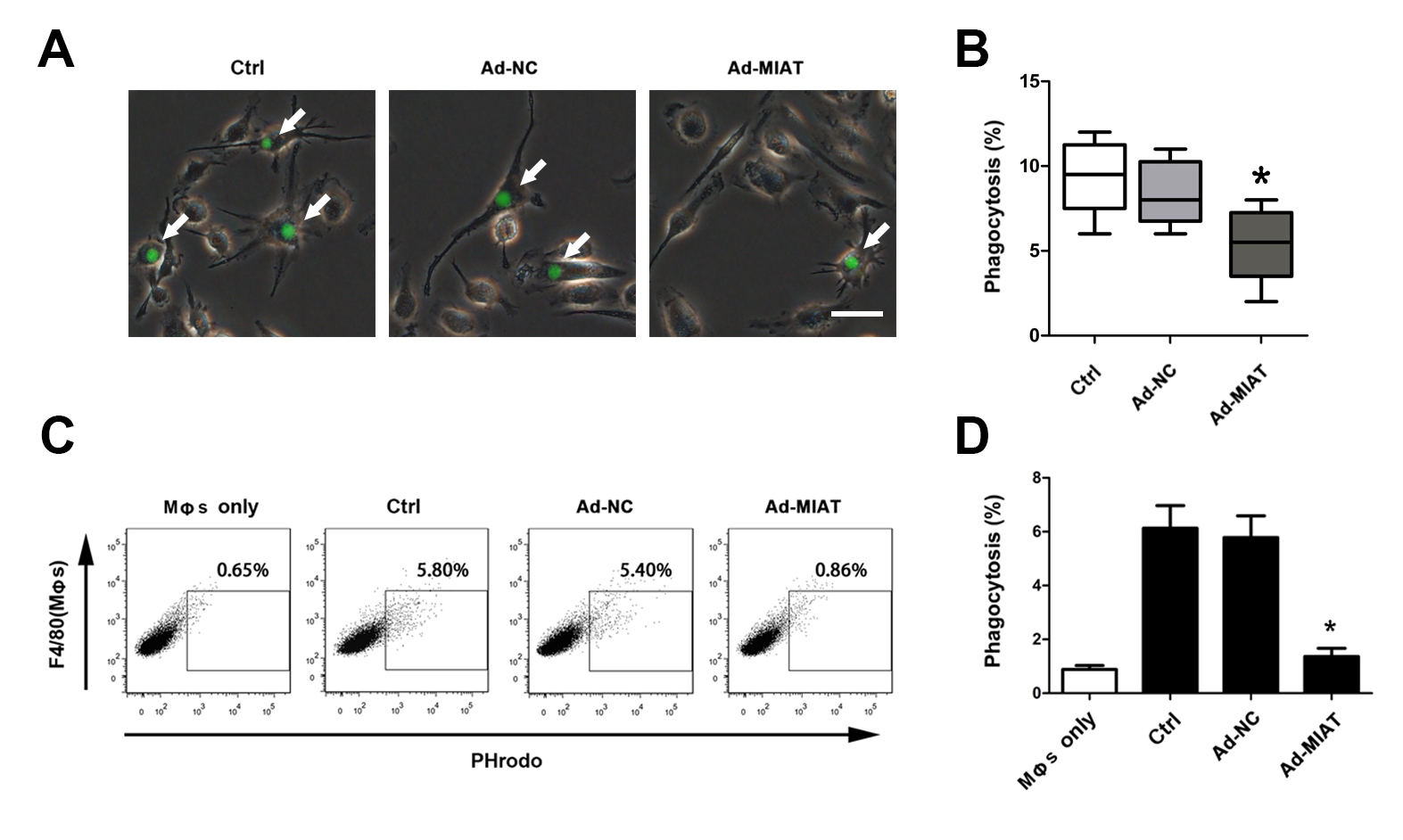

Supplement: Supplementary file 6 — Supplementary figure 6 [file 41419_2019_1409_MOESM6_ESM.jpg]
