# Supplementary material for: LncRNA MIAT sponges miR-149-5p to inhibit efferocytosis in advanced atherosclerosis through CD47 upregulation
Source: Cell Death Dis. 2019 Feb 12;10(2):138. doi: 10.1038/s41419-019-1409-4 (PMC6372637; doi:10.1038/s41419-019-1409-4)
Supplement: Supplementary file 7 — Supplementary table 1 [file 41419_2019_1409_MOESM7_ESM.doc]

**Supplementary table 1. Comparisons of baseline characteristics between Symptomatic and Asymptomatic AS group.**

| Variables | control group | Asymptomatic AS | Symptomatic AS | *P* |
| --- | --- | --- | --- | --- |
| n= | 20 | 18 | 20 |  |
| Symptoms |  |  |  |  |
| Stroke n= (in %) | n.a. | n.a. | 16(80.00%) | n.a. |
| TIA n= (in %) | n.a. | n.a. | 4(20.00%) | n.a. |
| Age (years) | 63.55±10.47 | 65.67±9.40 | 69.05±8.36 | 0.188 |
| Male gender n= (in %) | 9(45.00%) | 12(66.67%) | 13(65.00%) | 0.310 |
| Hypertension n= (in %) | 6(30.00%) | 11(61.11%) | 10(50.00%) | 0.147 |
| Hyperlipidemia n= (in %) | 7(35.00%) | 5(27.78%) | 12(60.00%) | 0.077 |
| Diabetes mellitus n= (in %) | 5(25.00%) | 7(38.89%) | 6(30.00%) | 0.648 |
| Coronary artery disease n= (in %) | n.a. | 1(5.56%) | 2(10.00%) | 0.359 |
| Current smoking n= (in %) | 6(30.00%) | 10(55.56%) | 12(60.00%) | 0.125 |
| Pulmonary disease n= (in %) | n.a. | n.a. | 1(5.00%) | n.a. |
| Previous PCI n= (in %) | n.a. | n.a. | 1(5.00%) | n.a. |
| Alcohol intake n= (in %) | 6(30.00%) | 7(38.89%) | 9(45.00%) | 0.619 |
| Hyperhomocysteinemia n= (in %) | 4(20.00%) | 6(33.33%) | 4(20.00%) | 0.547 |
| Abdominal obesity n= (in %) | 3(15.00%) | 6(33.33%) | 7(35.00%) | 0.296 |
