# Supplementary material for: LncRNA MIAT sponges miR-149-5p to inhibit efferocytosis in advanced atherosclerosis through CD47 upregulation
Source: Cell Death Dis. 2019 Feb 12;10(2):138. doi: 10.1038/s41419-019-1409-4 (PMC6372637; doi:10.1038/s41419-019-1409-4)
Supplement: Supplementary file 8 — supplemental figure legends [file 41419_2019_1409_MOESM8_ESM.docx]

**Supplementary Figure legends**

**Supplementary figure 1. MIAT was expressed in a few SMCs in atherosclerotic lesions.** RNA-fluorescent in situ hybridization (FISH) for MIAT (red) and immunostaining for α-SMA (green) was performed on frozen aortic root sections from 16-week HFD-fed ApoE^-/-^ mice. Stars denote macrophages expressing MIAT. The stars indicate ‘free’ apoptotic bodies. (Scale bar = 50μm).

**Supplementary figure 2. Quantification of plasma lipid profile from PBS-treated, Scr shRNA-treated, and MIAT shRNA-treated** **ApoE^-/-^ mice. A–D** The line graph shows the lipid profiles, including TC, TG, LDL, and HDL concentrations, at different times. **E** The line graphs show the weight of the mice at different times.

**Supplementary figure 3.** **Quantification of atherosclerotic lesions in PBS-treated and Scr shRNA-treated** **ApoE^-/-^ mice. A, B** ORO analysis of the percentage of lesion area to total aortic area in the PBS-treated and Scr shRNA-treated ApoE^-/-^ mice (n = 9/group). (Scale bar = 2mm). **C, D** ORO analysis of the relative lesion area of aortic root area in the PBS-treated and Scr shRNA-treated ApoE^-/-^ mice (n = 9/group). (Scale bar = 200μm).

**Supplementary figure 4. Ox-LDL treatment induced macrophage apoptosis.** Western blot analysis of Bax, cleaved caspase 3, caspase 3, and GAPDH expression in ox-LDL-treated Raw264.7 cells at the indicated doses (0, 25, 50, 100, 150 μg/mL) (**P＜0.05*, n = 6/group).

**Supplementary figure 5. Quantification of silence efficiency and macrophage apoptosis after siRNA transfection. A** qRT-PCR showed that MIAT expression in Raw264.7 cells was significantly down-regulated after transfection with siRNAs and si-MIAT-3 demonstrated the highest efficacy (**P*＜0.05, *^#^P*＜0.01, si-MIAT-1 or si-MIAT-3 vs. Ctrl, n = 6/group). **B** Annexin V-FITC and propidium iodide (PI) staining by flow cytometry assay showed that knockdown of MIAT by siRNA transfection did not affect apoptosis in Raw264.7 cells.

**Supplementary figure 6. Over-expression of MIAT decreased phagocytosis in vitro**. A Raw264.7 cells transfected with or without Ad-MIAT after ox-LDL treatment (150 μg/mL) were fluorescently labeled green using CFSE and incubated with BMDM for 2 h and then examined by fluorescence microscopy. Arrows indicate BMDM-derived macrophages containing phagocytosed apoptotic Raw264.7 cells. (Scale bar = 50 μm). B The phagocytic index (number of target cells ingested per 100 macrophages) was determined for the indicated cell lines (**P＜0.05*, Ad-MIAT vs. Ad-NC or Ctrl, n = 6/group). C Same as A, Raw264.7 cells transfected with or without Ad-MIAT after ox-LDL treatment (150 μg/mL) were fluorescently labeled green using pHrodo and incubated with BMDM for 2 h and then stained with APC-conjugated anti-F4/80. The stained cells were analyzed by flow cytometry. D The phagocytosis efficiency was determined from the percentage of F4/80+ cells containing pHrodo-derived green fluorescence (**P＜0.05*, Ad-MIAT vs. Ad-NC or Ctrl, n = 6/group).

**Supplementary table 1. Comparisons of baseline characteristics between symptomatic and asymptomatic AS group.**
